# Silver nanoparticle based antibacterial methacrylate hydrogels potential for bone graft applications

**DOI:** 10.1016/j.msec.2015.02.002

**Published:** 2015-05-01

**Authors:** M. Isabel González-Sánchez, Stefano Perni, Giacomo Tommasi, Nathanael Glyn Morris, Karl Hawkins, Enrique López-Cabarcos, Polina Prokopovich

**Affiliations:** a Department of Physical Chemistry, School of Industrial Engineering, Castilla-La Mancha University, Albacete, Spain; b Department of Physical Chemistry II, Complutense University of Madrid, Madrid, Spain; c School of Pharmacy and Pharmaceutical Sciences, Cardiff University, Cardiff, UK; d Department of Biological Engineering, Massachusetts Institute of Technology, Cambridge, USA; e Centre of Nanohealth, Institute of Life Sciences, Swansea University, Swansea, UK

**Keywords:** Hydrogels, Silver, Nanoparticles, Infections, *Staphylococcus epidermidis*, Methicillin-resistant *Staphylococcus aureus* (MRSA)

## Abstract

Infections are frequent and very undesired occurrences after orthopedic procedures; furthermore, the growing concern caused by the rise in antibiotic resistance is progressively dwindling the efficacy of such drugs. Artificial bone graft materials could solve some of the problems associated with the gold standard use of natural bone graft such as limited bone material, pain at the donor site and rejections if donor tissue is used. We have previously described new acrylate base nanocomposite hydrogels as bone graft materials. In the present paper, we describe the integration of silver nanoparticles in the polymeric mineralized biomaterial to provide non-antibiotic antibacterial activity against *Staphylococcus epidermidis* and Methicillin-resistant *Staphylococcus aureus*. Two different crosslinking degrees were tested and the silver nanoparticles were integrated into the composite matrix by means of three different methods: entrapment in the polymeric hydrogel before the mineralization; diffusion during the process of calcium phosphate crystallization and adsorption post-mineralization. The latter being generally the most effective method of encapsulation; however, the adsorption of silver nanoparticles inside the pores of the biomaterial led to a decreasing antibacterial activity for adsorption time longer than 2 days.

## Highlights

• Acrylate based hydrogels were prepared.
• Hydrogels were mineralized through reaction diffusion.
• Silver nanoparticles were encapsulated in different ways.
• Nanoparticle adsorption after mineralization was the most effective antibacterial method.

## 1 Introduction

Hydrogels are suitable for a variety of applications in the pharmaceutical and medical industry. They have been used in ophthalmology, for drug delivery, orthopedics and medical devices [1], [2], [3]. Among their characteristics are: soft and rubbery consistence, excellent biocompatibility and high permeability to oxygen, nutrients and other water soluble metabolites. All these characteristics make them particularly attractive as scaffolds in tissue engineering applications [4]. Very recently hydrogels have been used as a model to study biomineralization since these processes take place in gelling environments [5]. Biomineralization of an organic matrix has been an important topic in bone–tissue engineering [6], [7], [8], [9] as a possible process to prepare hybrid biomaterials for orthopedic tissue engineering where three-dimensional biomimetic mineralization is highly desired [10].

Hydrogels based on methacrylates are biocompatible, non-toxic, and non-immuno-reactive and their porosity is easily controllable [11]. After the pioneering work in the 1960s [12], methacrylate hydrogels have been applied in drug delivery systems, tissue regeneration, contact lenses and synthetic membranes for biosensors [13], [14], [15], [16], [17], [18]. Very recently hydrogels based on methacrylates have been also used as tissue expanders in dentistry clinics [19].

**Fig. 1:**
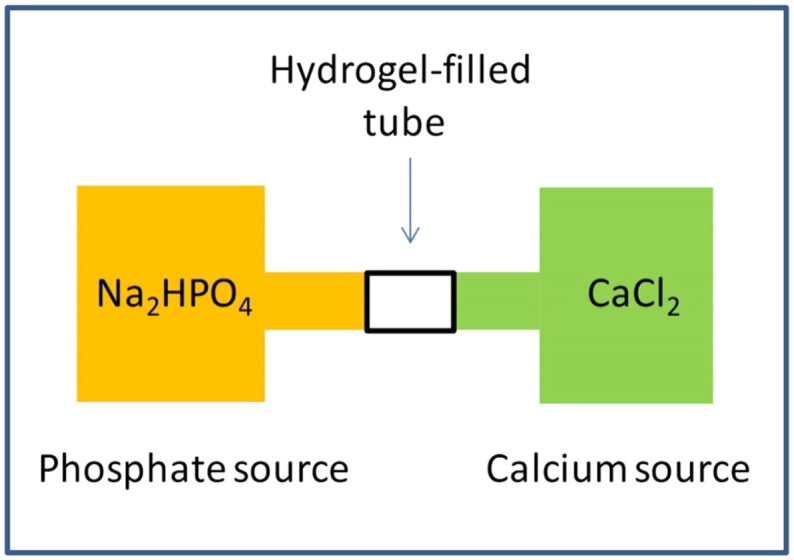
Representation of the reaction–diffusion process to produce calcium phosphate nanoparticles inside the hydrogel matrix.

We lately synthesized a copolymer hydrogel using as monomers PEG methyl ether methacrylate (PEGMEM) and (dimethylamino)ethyl methacrylate (DEM) [20]. Using Na_2_HPO_4_ and CaCl_2_, we were able to produce brushite microparticles in situ using the gel network as a microreactor for microparticle formation by means of a reaction–diffusion process (Fig. 1). In this process, phosphate and calcium ions migrate from the opposite sides of the gel and when they encounter the insoluble calcium phosphate particles form and precipitate. This composite hydrogels could be used in medical and dentistry applications as artificial bone graft material because of the osseconductive properties provided by the calcium phosphate microparticles homogeneously distributed in the gel matrix.

Infections associated with medical implants are becoming increasingly common and result in significant morbidity and, in some cases, mortality [21]. Very frequently biofilms, bacteria that attach to surfaces and aggregate in a hydrated polymeric matrix, are associated to infections because of their high resistance to antibacterial drugs [22]. Thus the synthesis of hydrogels with bactericidal properties has special interest. Some authors have been able to mix bactericidal agents into hydrogels [23], [24]. Another promising approach is the addition of metal nanoparticles as alternative to antibiotics because of the increasing bacteria population exhibiting resistance to these drugs consequently reducing their applicability [25], [26]. Silver nanoparticles have been shown effective to combat bacteria, viruses and eukaryotic micro-organisms [27], [28]. Besides, silver nanoparticles are also reported to possess anti-inflammatory [29] and anti-angiogenic activity [30], which makes these nanoparticles suited for medical purposes. Various synthetic routes have been developed to prepare silver nanoparticles and the conditions are responsible for the size, shape and surface charge of the resulting nanomaterials [31], [32], [33].

In this work, we examined ways to encapsulate silver nanoparticles in methacrylate hydrogels containing calcium phosphate to obtain a new multifunctional biomaterial that could be applied as a synthetic bone draft since it combines homogeneous 3D-biomineralization and antibacterial properties.

## 2 Materials and methods

### 2.1 Chemicals

Polyethylene glycol methyl ether methacrylate with average Mn = 300 (PEGMEM), 2-dimethylamino ethyl methacrylate (DEM), N,N′-methylenebis(acrylamide) (BIS), silver nitrate and citric acid were purchased from Sigma Aldrich. The initiator ammonium persulfate (APS) was purchased from Fluka (Spain). Na_2_HPO_4_·12H_2_O and CaCl_2_ were purchased from Panreac quimica SAU (Spain). All chemicals were reagent grade and used as received.

### 2.2 Synthesis of the silver nanoparticles

Silver colloids were prepared by the reduction of AgNO_3_ with citrate at near-boiling temperature. 125 ml of silver nitrate solution 1 mM was heated and as soon as boiling commenced, 5 ml of 1% sodium citrate solution were added. Heating was continued until a color change from uncolored to pale yellow was evident. Then the solution was removed from the heating element and stirred until it had cooled down to room temperature.

### 2.3 Silver nanoparticle characterisation

UV–vis spectra (350 – 600 nm, 1 nm resolution) of the conjugated nanoparticles dispersed in PBS (1 mg/ml) were recorded in 1 cm quartz cells with a U-3000 Hitachi, UV–vis spectrometer. For transmission electron microscopy (TEM) characterization a 4 µl droplet of nanoparticle suspension was placed on a plain carbon-coated copper TEM grid and allowed to evaporate in air under ambient laboratory conditions for several hours. Bright field TEM images were obtained using a TEM (Philips CM12, FEI Ltd, UK) operating at 80 kV fitted with an X-ray microanalysis detector (EM-400 Detecting Unit, EDAX UK) utilizing EDAX's Genesis software. Typical magnification of the images was × 100,000. Images were recorded using a SIS MegaView III digital camera (SIS Analytics, Germany) and analyzed with the software ImageJ.

### 2.4 Synthesis of the gel

The gels were prepared by adding 2 ml PEGMM (0.0065 mol) and 1 ml of DEM (0.0065 mol) to 5 ml of deionized water at room temperature. Subsequently, appropriate amounts of BIS and 1 mg/ml APS were added under stirring and finally the mixture was allowed to gel. It generally took around 25 min for a firm gel to form. After obtaining the gels, they were dialyzed in ultrapure water at room temperature for two weeks to remove the excess monomers and unwanted reaction products.

To obtain the hydrogel with silver nanoparticles entrapped, 5 ml of silver nanoparticle suspension at two different concentrations, 1 mM and 0.5 mM in water, was used instead of deionized water. The rest of the process was exactly the same.

### 2.5 Reaction diffusion experiments

A piece of the swelled gel was inserted in the middle of a plastic tube (diameter: 1 cm; length: 7 cm) connected to both sides with silicone tubes that ended each one in a bottle (500 ml) containing an aqueous solution of either CaCl_2_ (20 mM) or Na_2_HPO_4_ (20 mM), thus insuring a reservoir that guaranteed the continuous diffusion of the reactants through the gel. The reaction proceeded at room temperature for one week.

One of the methods of integration silver nanoparticles used in this work consisted in diluting the phosphate with the silver nanoparticle suspension (1 and 0.5 mM) and operating as described above.

### 2.6 Adsorption of silver nanoparticles in the hydrogels

Mineralized hydrogels were immersed in silver nanoparticle suspensions (5 ml) at two concentrations: as obtained from the synthesis (1 mM) and diluted 1:1 in distilled water (0.5 mM). The gels were stored at room temperature for up to 7 days. Every day a set of samples was removed from the silver nanoparticle suspension and rinsed in sterile PBS prior to further testing.

### 2.7 Silver content determination

Gel pieces of known weight were placed in glass bottles containing 5 ml of aqua regia (HCl:HNO_3_ 3:1); the bottles were sealed and stored at room temperature until the gels were completely dissolved. The silver ion content in the dissolved acid solution was determined by inductively coupled plasma–mass spectroscopy (ICP–MS) analysis (Optima 2100DV OES; Perkin Elmer, Waltham, MA, USA) against the Primar 28 element standard.

### 2.8 Bacterial species

The bacteria used were Methicillin-resistant *Staphylococcus aureus* (MRSA) (NCTC12493) and *Staphylococcus epidermidis* (RP62a). Bacteria were maintained by sub-culturing on Brain Heart Infusion (BHI) agar (Oxoid, Basingstoke, UK) and storing plates at 4 °C for no more than a week. For experimental purposes, bacteria were grown aerobically in 10 ml BHI broth (Oxoid, Basingstoke, UK) statically at 37 °C for 24 h.

### 2.9 Antibacterial assay

The antibacterial properties of the hydrogels containing silver nanoparticles were determined through the protocol developed by Berchet et al. [34] and widely employed [35], [36], [37]. Briefly, 500 μl of bacterial suspension was added on the hydrogel sample (diameter: 1 cm, thickness: 2 mm) contained in a 24 well plate; the plate was subsequently incubated aerobically at 37 °C for 1 h; the microbial suspension was then removed and the gel rinsed three times with sterile PBS. 500 μl of BHI diluted with PBS (1:10) was added in the well and the plate incubated at 37 °C for 24 h. An aliquot (50 μl) from each well was transferred in a 100 well plate (Bioscreen, Finland) containing 100 μl of fresh BHI broth. The plate was placed in a plate reader (Bioscreen, Finland) and the growth curves in each well recorded through optical density at 600 nm over a period of 24 h at 15 min intervals. The lag phase of each growth curve was calculated through fitting with the Baranyi–Robert model.

Experiments were performed in triplicates and from three independent cultures giving a total of 9 growth curves for each bacterium on each material.

The control samples for the entrapment during the polymerization step and their addition in the solution used for the mineralization consisted of the same hydrogel (cross-linking percentage) where solutions not containing silver nanoparticles were used during the corresponding phase; they are described in the text 0% Ag. For the absorption method, the control samples were hydrogels not exposed to nanoparticles and they are described as absorbance time 0 day.

### 2.10 In vitro cytotoxicity studies

Osteoblast cells (MC-3T3) were cultured in Dulbecco's Modified Eagle's Medium supplemented with fetal bovine serum (10% v/v); cells were incubated at 37 °C in a humidified atmosphere with 5% CO_2_. Cells were grown till confluence was reached, washed twice with sterile PBS and detached with trypsin.

Samples were placed in 24-well plate with 500 μl of osteoblast cell suspension and incubated with the composite gel at 37 °C in a humidified atmosphere with 5% CO_2_. After 2 days, the medium was removed and 1 ml fresh medium without red phenol was added. Osteoblast cell viability was assessed using the MTT assay kit (Invitrogen, Paisley, UK) with 20 μl of the reagent solution, prepared according to the manufacturer instructions, added to each well. After incubation for 2 h at 37 °C in a humidified atmosphere with 5% CO_2_ all the solutions were removed and the MTT solubilization solution was added. When full dissolution of the crystals occurred, 100 μl of liquid was transferred to a 96-well plate where the absorbance of each sample was read at 570 nm.

### 2.11 Rheological properties of gels

The swelled gels (after dialysis, reaction–diffusion mineralization and adsorption in silver nanoparticle solution) were cut into a circle through a stamp of 25 mm diameter and loaded into the rheometer (ARES-G2 Rheometer (TA Instruments)). Rheological tests were performed at 37 °C and stainless steel parallel plates (25 mm diameter) were employed to sandwich the material at a constant but low normal force (4 N). G′ was monitored as the material equilibrated and as the gap reduced. When G′ became constant, it was assumed that the material was appropriately in contact with the plates; tests were conducted in the frequency range from 0.01 to 10 Hz. All measurements were conducted at a strain of 0.1%, which was within the linear visco-elastic range of the material, as confirmed by a strain sweep and the absence of a third harmonic response.

### 2.12 Statistical methods

In order to assess the antibacterial activity of the hydrogels, the lag phase durations obtained from different preparation methods were compared using ANOVA followed by post hoc Tukey's test for individual pairs of data sets.

## 3 Results and discussion

### 3.1 Characterization of the silver nanoparticles synthesized

**Fig. 2:**
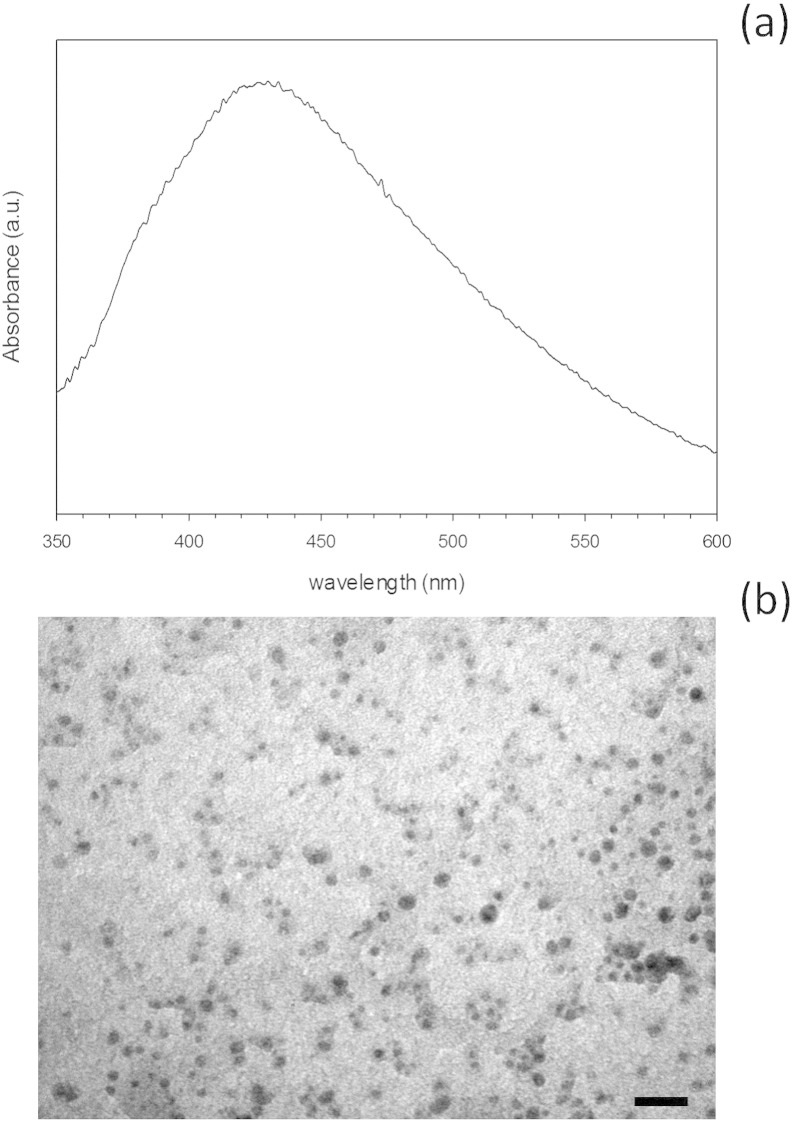
UV–vis spectrum (a) and TEM image (Bar equals to 250 nm) (b) of silver nanoparticles.

Silver nanoparticles were synthesized by citrate reduction of silver ions at near-boiling temperature. The UV–vis spectra of the nanoparticle suspension presented an absorbance maximum around 440–460 nm (Fig. 2a) typical of silver nanoparticles [27], [32], [33], [35], [38], [39]. This method yielded relatively large silver nanoparticles with a diameter of 60–80 nm and rounded shape as seen through TEM (Fig. 2b).

### 3.2 Silver nanoparticle encapsulation in hydrogels

The silver nanoparticles were incorporated in our composite with the aim to provide the antibacterial properties. The incorporation of the nanoparticles was achieved by means of different methods related to the different steps of the hydrogel preparation and the results were compared. The methods were: 1) mixing the silver NPs with the monomers before the gel synthesis process; 2) diluting sodium phosphate in the nanoparticle suspension to integrate them during the diffusion reaction experiment and 3) by means of simple adsorption. The antibacterial and cytotoxicity properties of these new silver nanoparticle-composite gels were analyzed. In all the cases, the antibacterial properties of the hydrogel with nanoparticles were determined through the apparent lag phase and growth rate of the MRSA and *S. epidermidis* cells detached from the hydrogel samples. These bacterial species were selected as they are the main sources of orthopedic infections [40], [41]; furthermore, the *S. epidermidis* strain used in this work is also already resistant to commonly used antibiotics such as gentamicin [42].

This indirect method of determining the microbial load of the samples is based on the correlation between cell concentration in the broth and the duration of the apparent lag phase of the growth curve determined through optical density [34]. The growth rate is, instead, related to the physiological state of the bacteria after exposure to the bactericidal agent [35], [36].

The direct mixing of the antibacterial drug [24] or of nanoparticles [43], [44] in the hydrogel formation mixture is the most common form of antibacterial hydrogel preparation; however as other steps in the hydrogel formation process are available, they can also be exploited for such purpose. Alternatively, in situ nanoparticle formation has been achieved [45].

#### 3.2.1 Silver nanoparticles entrapped in the composite

Firstly, we tried to provide antibacterial activity to the nanocomposite hydrogels by encapsulating the silver nanoparticles directly during the synthesis. The amount of silver present in the hydrogels prepared in this way after the mineralization phase was independent from the concentration of silver in the solution and the percentage of the cross-linking agent (Table 1).

**Fig. 3:**
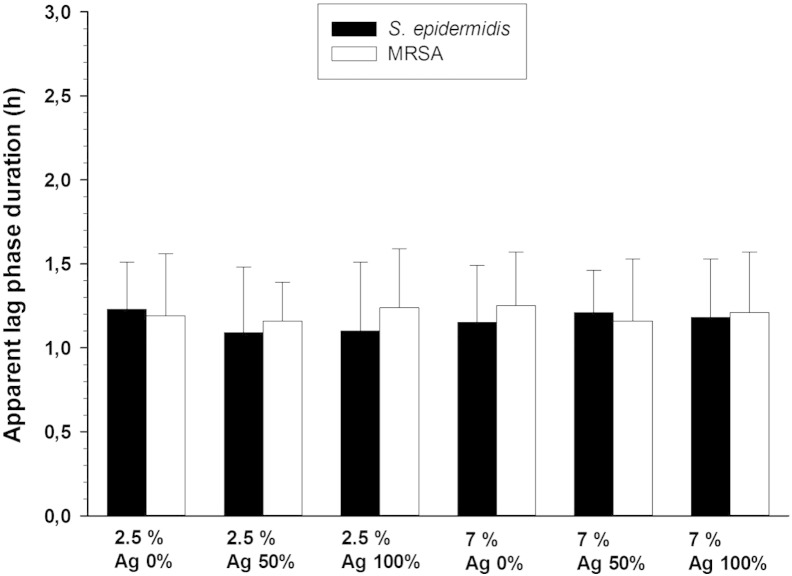
Apparent lag phase duration of MRSA and *Staphylococcus epidermidis* on hydrogels with different crosslinking degrees with AgNPs encapsulated within the gels during polymerization.

The apparent lag phase obtained for hydrogels with 2.5% and 7% is shown in Fig. 3; samples containing silver nanoparticles had the same apparent lag phase duration than controls (without silver nanoparticles) (p > 0.05) meaning that no antibacterial effect was achieved by this method of encapsulation.

The dialysis phase did not result in the total removal of silver from the hydrogels (Table 1); furthermore we have checked that the entrapment of the silver nanoparticles inside the hydrogel without calcium phosphate (before mineralization through reaction–diffusion) had antibacterial activity (data not shown). Therefore, the precipitation of calcium phosphate is likely to be responsible for the loss of the antibacterial properties as it could prevent the silver ion flow across the surface of the composite. This is probably due to the formation of a film of calcium phosphate and/or silver phosphate.

#### 3.2.2 Diffusion of the silver nanoparticles into the composite

Because of the inefficacy of the direct mixing of the silver nanoparticles in the hydrogels during the polymerization phase, the encapsulation of the antibacterial nanoparticles was attempted during the mineralization phase. Sodium dihydrogen phosphate was diluted in the silver nanoparticle suspension, instead of distilled water, at the same concentration used previously during the mineralization step (the color of the suspension changes from yellow to green). In this way the silver nanoparticles were introduced in the hydrogels at the same time as the calcium phosphate crystals were formed. The preparation of the other solution (CaCl_2_) with silver nanoparticles was not possible because of the precipitation of AgCl.

**Fig. 4:**
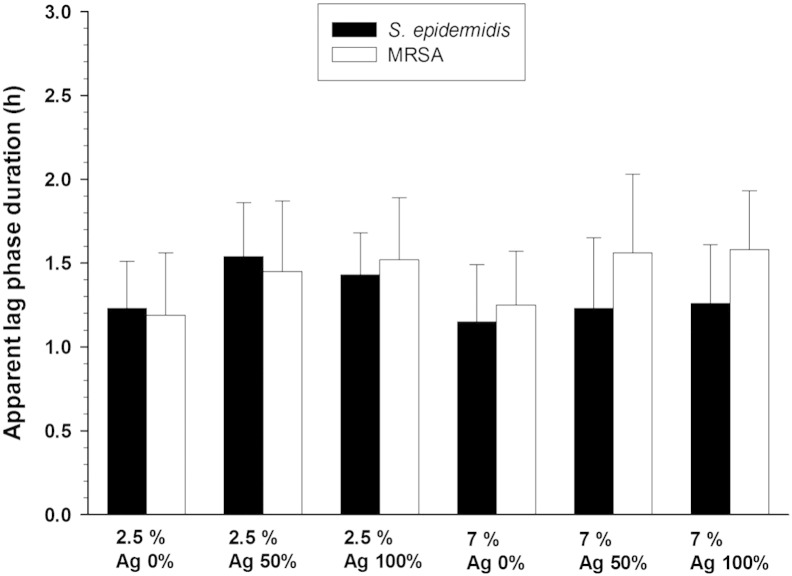
Apparent lag phase duration of MRSA and *Staphylococcus epidermidis* on hydrogels with different crosslinking degrees with embedded AgNPs through diffusion during mineralization.

Control samples (without the nanoparticles) showed the OD at 600 nm of the bacterial suspension started increasing after about 1.2–1.3 h for both 2.5% and 7% crosslinking concentrations (Fig. 4); a small antibacterial effect was appreciated since the apparent lag phase duration increased about 0.2–0.3 h. Such bactericidal effect was not statistically significant (p > 0.05).

The precipitation of the Ag^+^ with the phosphate ions (Ag_3_PO_4_ has a solubility constant product Kps = 8.89 × 10^− 17^) [46] in the solution used during the reaction–diffusion process could be dragging the nanoparticles to the bottom of the bottle reducing their availability towards the composite gel.

#### 3.2.3 Adsorption of silver nanoparticles into the composite

The last method tested was adsorption by putting in contact the already mineralized hydrogels with silver nanoparticle suspension. Adsorption of silver nanoparticles in the composites was carried out at different contact times (daily increase from 1 to 6 days).

Hydrogels prepared with 2.5% of cross-linker did not exhibit increasing amount of silver with prolonged exposure to silver nanoparticles (Table 2) regardless of the concentration of silver nanoparticles used. However, when the crosslinking agent was increased to 7% the concentration increased with time when a 0.5 mM silver nanoparticle suspension was used. No significant differences were recorded between the first 3 days of adsorption when a 1 mM nanoparticle suspension was employed (p > 0.05), after 6 days the quantity of silver present in the hydrogels was the same irrespectively to the crosslinking quantity and concentration of the silver nanoparticle suspension (p > 0.05).

**Fig. 5:**
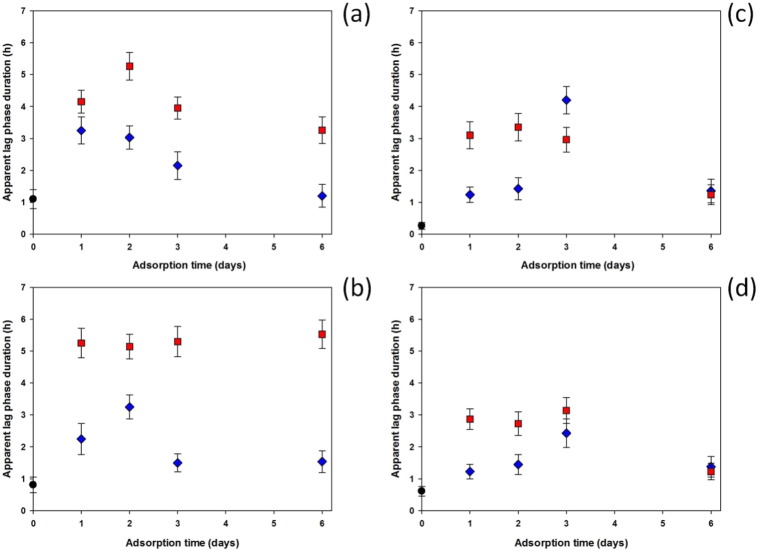
Apparent lag phase duration of *S. epidermidis* on (a) 2.5% crosslinking and (b) 7% crosslinking hydrogels and MRSA on (c) 2.5% crosslinking and (d) 7% crosslinking hydrogels as function of adsorption time of silver nanoparticles against samples without adsorption (controls). 1 mM silver NP solution,  0.5 mM silver NP solution,  control samples.

Fig. 5 shows the comparison of all the apparent lag phases from the antibacterial test for adsorption performed for different lengths of time. The cross-linking degree (2.5% and 7%) seemed not to affect the samples that were not exposed to nanoparticle adsorption (control or 0 days of adsorption), resulting in similar apparent lag phases in both cases. Samples in contact with 1 mM silver nanoparticle suspension had a better antibacterial effect than those in 0.5 mM silver nanoparticle suspension. Comparing the two different bacteria, a better effect against *S. epidermidis* than MRSA was observed. In general, the maximum effectiveness was obtained for samples that were in contact with silver nanoparticles for 2 days. Nanoparticle adsorption performed for longer than 2 days did not improve the antibacterial activity that decreased for samples that adsorbed silver nanoparticles for 3 and 4 days. Apparent lag phases for MRSA were similar to those of controls for samples in contact with the silver nanoparticle suspension for 6 days. The only exception was *S. epidermidis* on samples immersed in silver nanoparticles of 1 mM that showed a constant apparent lag phase independent from the duration of the adsorption on 7% cross-linked hydrogels and apparent lag phase longer than control on 2.5% cross-linked hydrogels.

The amount of nanoparticles embedded in the gels through adsorption is likely to result in nanoparticles mainly distributed on the outer surface of the gels after short period of time, when the adsorption was allowed to continue for longer periods of time nanoparticles could have migrated deeper in the hydrogel matrix as shown by the increasing amount of silver present in the matrix without significant antibacterial improvement when adsorption was performed for longer periods of time. Furthermore, the possible reaction of silver with phosphate, resulting in Ag_3_PO_4_, could also prevent the antibacterial activity of the nanocomposite gels.

In order to comprehend the influence of incorporation methods on antibacterial activity, the bactericidal mechanism of silver nanoparticles needs to be fully understood. Silver nanoparticle surface can release Ag^+^ so even simple colloid solutions contain three forms of silver: Ag solid, free Ag^+^ or its complexes and surface-adsorbed Ag^+^. The relative contribution of each type of silver to the antibacterial activity remained unclear. Xiu et al. [47] have stated that such activity is solely due to Ag^+^ release suggesting that the nanoparticles themselves do not affect the biological activity of microbes and thus the silver nanoparticles may serve as a vehicle to deliver Ag^+^ more effectively. However, several studies have shown that silver ions released from nanoparticles are not the sole reason for silver nanoparticle antibacterial activity [35], [36] and that silver nanoparticles may attach to the surface of the cell membrane disturbing permeability and respiration functions of the cells [48]. Hence, it appears that both silver ions and nanoparticles contribute to the overall bactericidal activity [49]. Our hydrogels exhibit amino groups due to one of the monomers, this could have increased the release of Ag^+^ in virtue of electrostatic repulsion. However, the relative high ion concentration of biological media is likely to screen these charges (electrostatic double layer) resulting in low zeta potential of the hydrogels.

The relative higher resistance of MRSA compared to *S. epidermidis* is not only strain dependent, but also appears to be influenced by the characteristic of the silver nanoparticles. For example, with oleic acid capped silver nanoparticles *S. epidermidis* RP62a is stronger than MRSA NCTC12493 [35], while with citrate capped silver nanoparticles used in this work this is reversed (Fig. 5). The different sizes and capping agents between these two types of silver nanoparticles highlight further the role of the geometrical and physico-chemical parameters of the nanomaterials on the biological responses they induce.

Gentamicin and Tobramycin are commonly used in orthopedic material such as PMMA bone cement because of their broad activity spectrum and stability to high temperature. However the reliance on antibiotics to fight infections is now seen as a short term solution in light of the rise and spread of bacteria cells not affected by such drugs. Antibiotic resistance is a major danger and the development of novel strategies to prevent/treat infections is urgently needed [25], [26] because new antibiotics are rarely discovered (the last new antibiotic class dates back to the 1980s) and it is widely accepted that it is only a matter of time before resistance is developed towards a new antibiotic. Moreover, it is important to prepare antibacterial drugs that are effective against already resistant cells not only unable to induce further resistance. We employed *S. epidermidis* RP62a because it is a well known strain involved in human infections and is one of the many *S. epidermidis* strains resistant to gentamicin [42], [50], [51]. From this stand point, the hydrogels containing AgNPs presented here appear a better proposition than antibiotic eluting hydrogels in virtue of their activity against gentamicin resistant strains and no reliance on the use of another antibiotic (such as: daptomycin) in combination with gentamicin [52].

### 3.3 Cytotoxicity of hydrogels containing silver nanoparticles

**Fig. 6:**
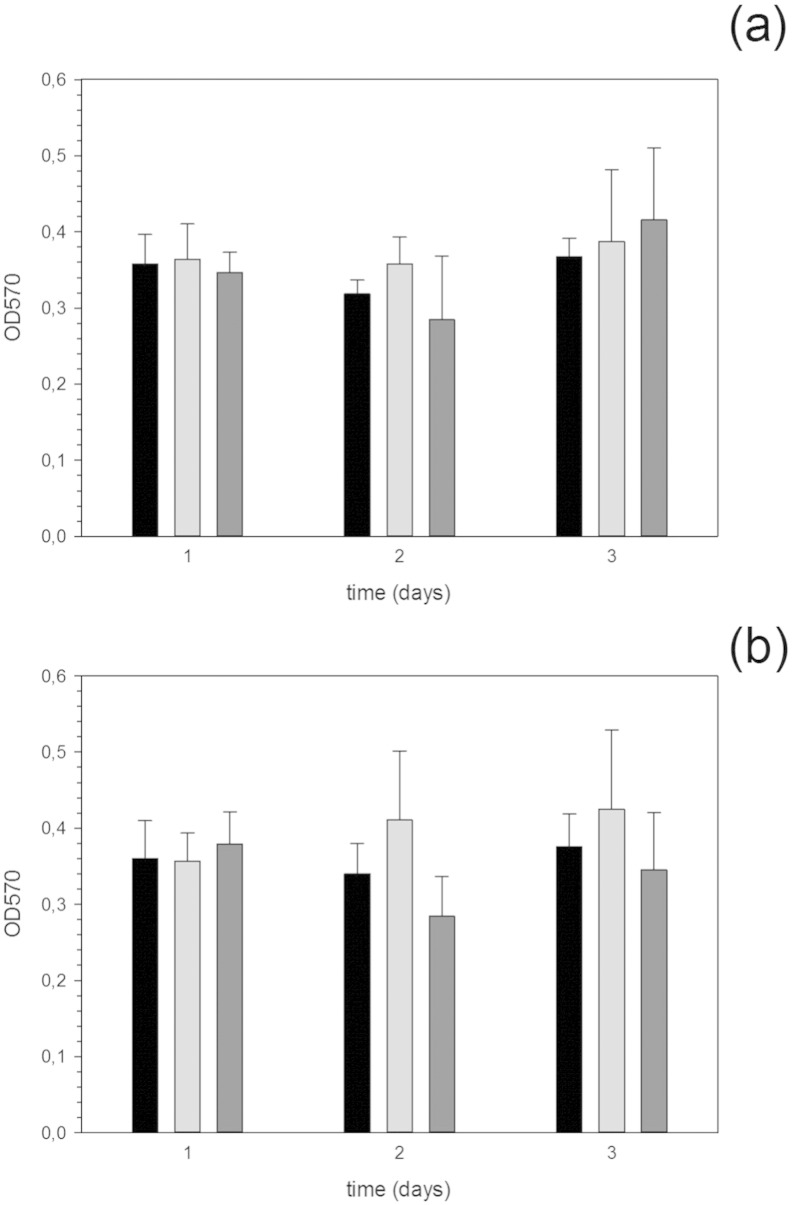
MTT assay of osteoblast activity after various contact time on hydrogels with 2.5% cross-linking (a) and 7% cross-linking (b) containing Ag nanoparticles through adsorption for various days. 0% (control),  0.5 mM Ag NP solution,  1 mM Ag NP solution.

The possibility of Ag nanoparticles having a cytotoxic effect was investigated through the MTT assay on osteoblast cells (Fig. 6). These cells have been used because osteoblasts are expected to grow directly on the implant surface in contact with bone. The results revealed that the presence of nanoparticles did not have a detrimental effect on the growth of osteoblast cells regardless of the cross-linking concentration and amount of nanoparticles used during the adsorption process. Such results were expected as generally silver nanoparticles do not exhibit cytotoxic properties.

### 3.4 Rheology of hydrogels containing silver nanoparticles

**Fig. 7:**
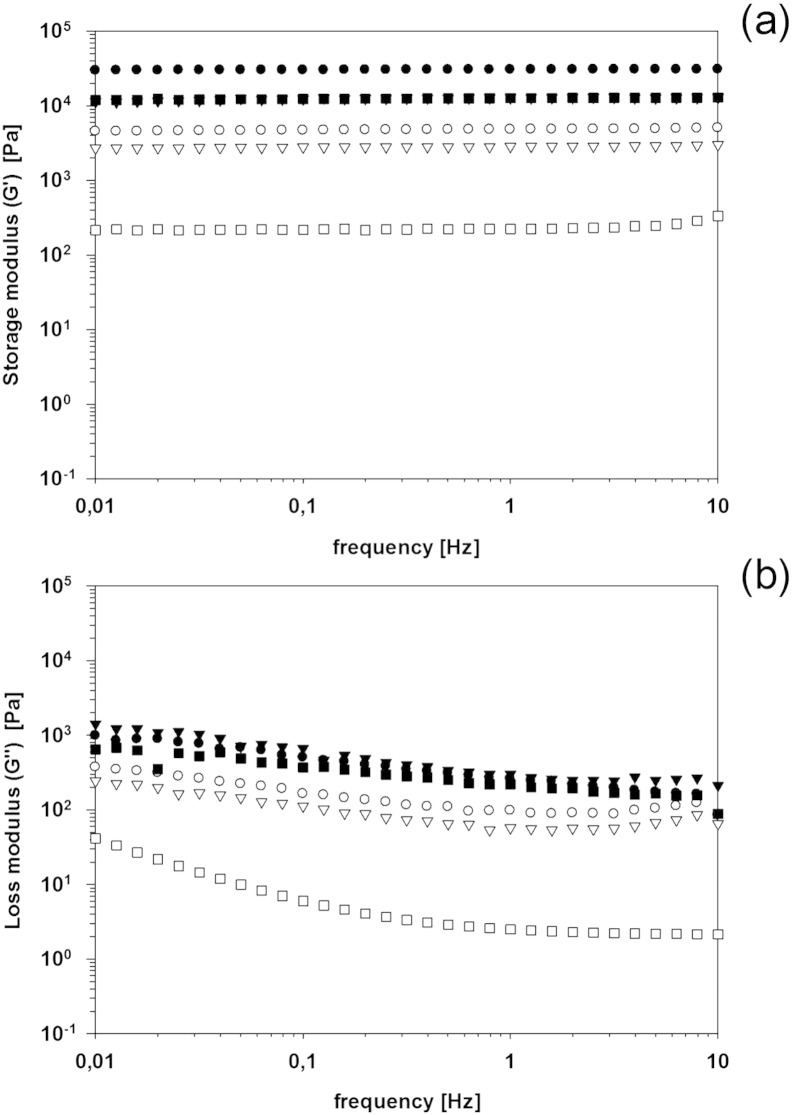
(a) Storage (*G′*) and (b) loss modulus (*G″*) of hydrogels with 7% (filled symbols) and 2.5% (empty symbols) of crosslinker prepared with aqueous solution of the silver nanoparticles. ● 0 (control), ▼ 0.5 mM, ■ 1 mM.

The rheological properties of hydrogels as function of the cross-linking agent percentage and the concentration of silver nanoparticles in the aqueous solution during polymerization are shown in Fig. 7. The elastic modulus (*G′*) did not change with frequency for all gels regardless of the concentration of the silver nanoparticles, however its value was dependent on both the percentage of the cross linking and the silver concentration (Fig. 7a). With pure distilled water, when 7% of cross linking agent was employed, *G′* was 30 KPa, while with 2.5% of BIS, *G′* was 5 KPa. On the other hand, *G″* did show a decreasing behavior with increasing frequency (Fig. 7b); with values of *G′* about 10 and 30 times *G″* at (0.1 Hz) for 7% and 2.5% crosslinking composition, respectively.

When silver nanoparticles were present in the polymerization solution the resulting hydrogels exhibited lower *G′* and *G″* than the corresponding hydrogels without nanoparticles. This effect was greater for hydrogels with 2.5% crosslinking than 7%, furthermore the concentration of silver in the solution did affect the latter hydrogels more than the former (Fig. 7).

**Fig. 8:**
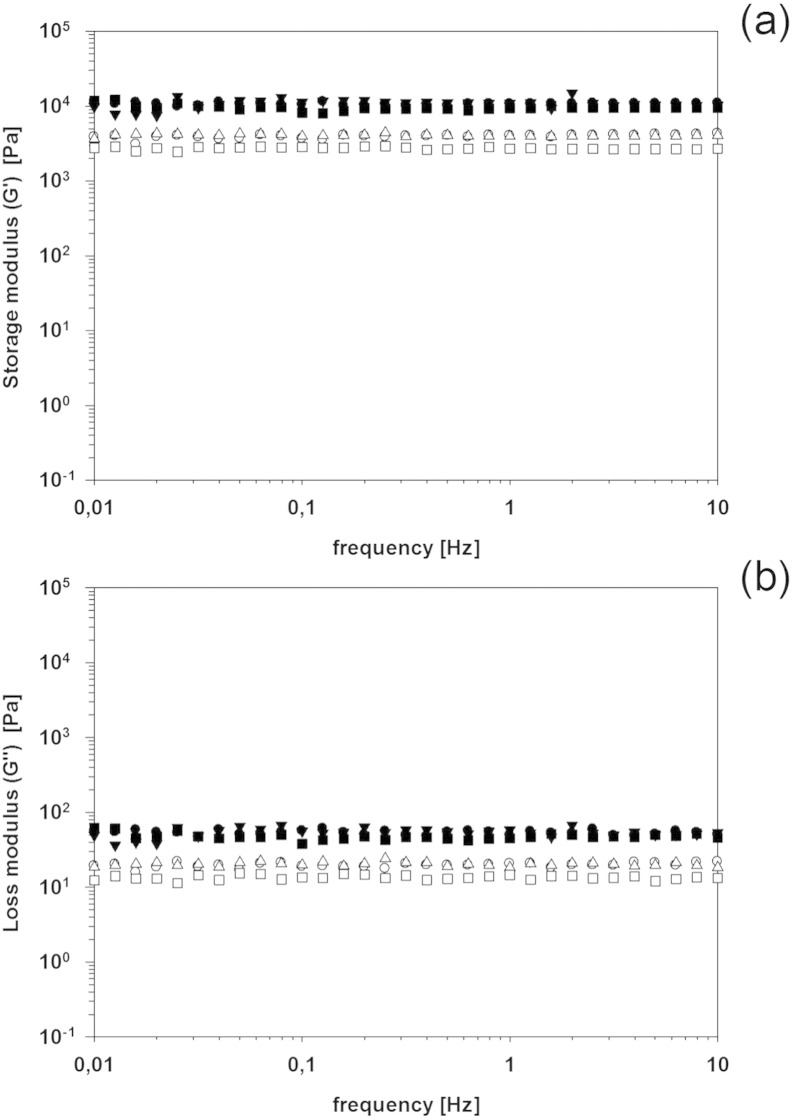
(a) Storage (*G′*) and (b) loss modulus (*G″*) of hydrogels with 7% (filled symbols) and 2.5% (empty symbols) of crosslinker after immersion in a solution silver nanoparticles (1 mM). ● after 1 day, ▼ after 2 days.

**Fig. 9:**
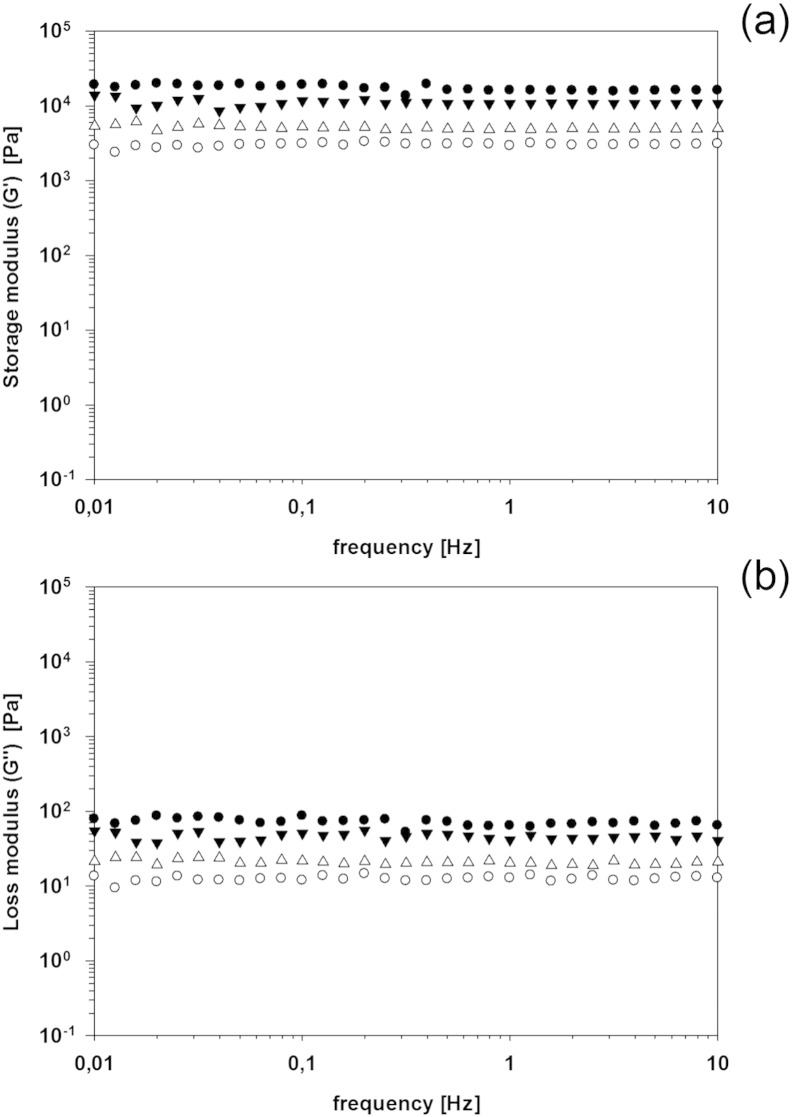
(a) Storage (*G′*) and (b) loss modulus (*G″*) of hydrogels with 7% (filled symbols) and 2.5% (empty symbols) of crosslinker after immersion in a solution silver nanoparticles (0.5 mM). ● after 1 day, ▼ after 2 days.

It is possible that the citrate ions present in the silver nanoparticle solution interfere with the polymerization reaction acting as radical scavenger as citrate ions are known antioxidant [53]. This activity would increase the level of un-reacted monomers and thus reduce the length of the polymer chains. Such phenomenon would explain the lower mechanical properties of the hydrogels when nanoparticles are present, instead of their enhancement as in a normal nanocomposite material. No particular impact on both G’ and G” was noticed when silver nanoparticles were embedded through adsorption post hydrogels mineralization (Fig. 8, Fig. 9) independently from the length of the adsorption.

Many orthopedic procedures, i.e., fixation of fractures caused by traumatic events or through osteoporosis, joint replacement, dental implants, and bone cancer treatment requiring bone augmentation as bone self regeneration properties can heal areas of only limited size; it is estimated that 2.2 million of orthopedic procedures worldwide employ bone grafts annually [54]. Although the use of real bone to fill bone voids (bone graft) is the gold standard technique because of the high osseoconductive and osseintegrative properties of bone, it exhibits numerous drawbacks such as: pain at the donor site and limited amount of bone available in case the bone graft is taken from the patient (autograft). Rejection is also a risk when the transplanted tissue is taken from a different patient (allograft) or animal (xenograft). In order to overcome these shortcomings a number of biomaterials (porous metal, bioactive glass, glass ceramics, calcium phosphate/sulfate polymers) have been developed as bone tissue engineering scaffolds [55], [56]. However, none of these exhibit completely satisfactory properties as they can exhibit good structural properties but they do not possess adequate osteoinductive, osteoconductive and osseointegrative properties [54]. Therefore the design of synthetic bone grafts that mimics the structure, composition and mechanical properties of bone, possesses good surgical handling and is cytocompatible remains a major challenge. The problems associated to bone graft are further aggravated by the possibility of infection occurring after surgery. For this reason the hydrogels presented here are simultaneously addressing the need for artificial bone graft materials capable of preventing/fighting infections and the use of non-antibiotic antibacterial agents, the later is major benefit compared to other works focusing on antibiotic release [57], [58], [59].

## 4 Conclusions

In order to provide antibacterial properties to the osseoconductive acrylate hydrogels we developed previously, the incorporation of silver nanoparticles has been performed using three different approaches.

The entrapment of the silver nanoparticles during the polymerization step and their addition in the solutions used for the mineralization of the hydrogel were performed without obtaining antibacterial properties. Only the adsorption of the nanoparticles on the biomineralized composite gel exhibited antibacterial activity. Such approach also did not negatively impact the cytotoxicity of the materials against osteoblasts and no detrimental consequences were observed on the rheological characteristics of the hydrogels.

The material presented here is one of the first examples of an acrylate multifunctional orthopedic hydrogel as it is simultaneously osseoconductive and non-antibiotic based antibacterial.

## Figures and Tables

**Table 1:** Silver content (μg Ag/g gel) in hydrogels after preparation using silver nanoparticle suspension (1 mM and 0.5 mM) in the polymerization mixture.

Cross-linking	Silver nanoparticle concentration	Silver content (μg Ag/g gel)
7%	1 mM	4.4 ± 0.2
	0.5 mM	4.5 ± 0.3
2.5%	1 mM	5.1 ± 0.3
	0.5 mM	5.4 ± 0.4

**Table 2:** Silver content (μg Ag/g gel) in hydrogels after adsorption in silver nanoparticle suspension (1 mM and 0.5 mM) for various times.

Adsorption (days)	Cross-linking			
7%		2.5%	
1 mM	0.5 mM	1 mM	0.5 mM
1	8.3 ± 0.3	5.8 ± 0.2	8.0 ± 0.2	9.4 ± 0.3
2	7.8 ± 0.3	8.9 ± 0.4	9.6 ± 0.4	9.1 ± 0.4
3	8.4 ± 0.3	10.4 ± 0.4	10.9 ± 0.3	10.6 ± 0.4
6	12.9 ± 0.4	13.8 ± 0.5	10.5 ± 0.4	10.4 ± 0.3
